# On the Nutrition of Muscles during Their Contraction

**Published:** 1852-07

**Authors:** E. Brown-Séquard

**Affiliations:** Paris


*On the Nutrition of Muscles during their Contraction.* By E. Brown-Sequard, M. D., of Paris.

It is generally admitted that when a muscle is in a state of powerful contraction, circulation, and consequently nutrition, are nearly arrested in it.

The well-known fact, that we are only able to maintain a permanent contraction in any of our muscles for a short time only, has been explained by a loss of strength occurring, from the supposed insufficiency of their nutrition. I have frequently performed a very simple experiment which proves that the cause of the rapid diminution of the power of our will, in that case, does not exist in the muscles themselves.

The experiment referred to has sometimes been made on my legs, sometimes on my arms; and it was conducted as follows: I took a weight in one of my hands, and kept my forearm in a state of flexion, so as to form with my arm an angle of only 25 or 30 degrees. In that condition some muscles, and more particularly the biceps, were in a state of permanent contraction. My ability to maintain my fore-arm in such a position, lasted between eight and twelve minutes. When I found it was completely impossible for me to keep my fore-arm in that position, an assistant applied the wires of an electro-magnetic machine to my shoulder and my fore-arm, so as to excite the biceps, and some other muscles. When, without any effort of my will, my fore-arm was maintained, nearly in the same position, during more than ten or even fifteen minutes.

After one or two minutes of galvanization, I occasionally tried again the action of the will, and I found that it was able to act anew.

If the explanation be true, that the muscle is not sufficiently nourished, and loses, in a great measure, its irritability during its contraction, and that for this reason the will becomes unable to maintain the contraction longer than a certain time, then the action of galvanism ought to be incapable of producing the contraction. If galvanism is able to act as it does, it is because the circulation of blood, the nutrition, and consequently the muscular irritability, have been very little diminished in the contracted muscles.

I have made another experiment, proving, also, that nutrition continues to take place in muscles during their contraction. If the communication established by the nerves, between the muscles of a mammal and its spinal marrow, is left entire; and if the circulation is completely stopped in that limb, by an amputation of the leg at the hip-joint, then it is found that the muscles of that leg, under the excitation of a powerful galvanic apparatus, lose their irritability after ten or fifteen minutes. On the contrary, if the same galvanic excitation is applied to the muscles of the other leg of the same animal, it is found that the irirritability remains a long time without a marked diminution, and that it cannot be completely exhausted. It diminishes little by little, but never disappears entirely. Therefore, it is evident that nutrition may take place in muscles during powerful contraction.

Four distinct organs are active in the case of a voluntary muscular contraction. 1st, the brain, i. e. the organ of the will; 2dly, the spinal marrow; 3dly, certain nerves; 4thly, certain muscles. Which of these is the one which is deficient in the case of my first experiment? It is generally admitted that it is the muscular irritability. My experiments prove that it is not so. Consequently, it remains to know in which of the three other organs exists the deficiency. It appears to me that it is in the brain, because a great many experiments have demonstrated that the nerves and the spinal marrow, when put strongly in action, remain very active during a long time, provided that the circulation of blood continues in them.

In saying that the action of the brain is deficient, I do not mean the action of the whole brain, but that of the part of that organ which is used in producing the contraction of these muscles, which are put in action.

From the preceding facts and reasonings I think I am justified in concluding:

1st. That it is the action of a part of the brain, and not the muscles, which is deficient, when our will is unable to maintain a permanent contraction in them.

2d. That circulation and nutrition are but little diminished in muscles strongly contracted.

